# Thoracic vertebral body erosion due to a perianeurysmal outpouching lesion after thoracic endovascular aortic repair: a case report and literature review

**DOI:** 10.1186/s12891-024-08265-5

**Published:** 2025-01-07

**Authors:** Hong Jin Kim, Joonghyun Ahn, Kee-Yong Ha, Dong-Gune Chang

**Affiliations:** 1 Spine Center and Department of Orthopedic Surgery, Inje University Sanggye Paik Hospital, College of Medicine, Inje University, 1342, Dongil-Ro, Seoul, Nowon-Gu 01757 Republic of Korea; 2 Department of Orthopedic Surgery, Kyung-in Regional Military Manpower Administration, Suwon, Gyeonggi-Do Korea; 3 Department of Orthopedic Surgery, Bucheon St. Mary’s Hospital, College of Medicine, The Catholic University of Korea, Bucheon, Korea; 4 Department of Orthopedic Surgery, Seoul St. Mary’s Hospital, College of Medicine, The Catholic University of Korea, Seoul, Korea

**Keywords:** Vertebral body erosion, Aortic arch aneurysm, Thoracic endovascular aortic repair, Perianeurysmal outpouching lesion

## Abstract

**Background:**

The safety of endovascular treatment, such as thoracic endovascular aortic repair (TEVAR), for a descending thoracic aortic aneurysm has been well-established, with a reported low postoperative mortality rate but higher incidences of long-term complications such as endo-leakage, device failure, and aneurysm-related death. Based on this, we report the first case of massive thoracic vertebral body erosion due to a perianeurysmal outpouching lesion after TEVAR.

**Case presentation:**

A 77-year-old female with a history of TEVAR due to descending thoracic aortic arch aneurysm 4 years ago was referred from the cardiovascular clinic to the spine center. The patient presented with persisting back pain, which began 3 years after TEVAR and progressively worsened. Physical examination was notable for tenderness in the upper thoracic region without any neurological deficits. Computed tomography of the aorta and thoracic spine showed bony erosion into the T5–T7 vertebral bodies. Magnetic resonance imaging of the thoracic spine confirmed a perianeurysmal outpouching lesion eroding into the T5–T7 vertebral bodies due to pulsating pressure. We performed the posterior instrumented fusion from T3 to T9 at the thoracic spine and TEVAR at remnant endo-leakage lesions.

**Conclusions:**

Since the progression of such a condition can have a catastrophic outcome, and because the treatment options vary, serial follow-up through an interdisciplinary approach is important in cases with a high index of suspicion of a perianeurysmal outpouching lesion.

## Background

Thoracic endovascular aortic repair (TEVAR) significantly contributes to surgery for aortic arch aneurysms by decreasing mortality and postoperative complication rates [1, 2]. The safety of endovascular treatment for a descending thoracic aortic aneurysm has been well-established, with a reported low postoperative mortality rate. However, higher incidences of long-term complications, including endo-leakage, device failure, and aneurysm-related death, have also been documented [2]. A thoracic vertebral body erosion secondary to an aortic aneurysm is a rare complication and has been described only in prior case reports [3–9]. Furthermore, to the best of our knowledge, it is a very rare case of thoracic vertebra body erosion after TEVAR, considering previously reported. Thus, we present and discuss the rare case of thoracic vertebral body erosion due to a perianeurysmal outpouching lesion after TEVAR.

## Case presentation

**Fig. 1:**
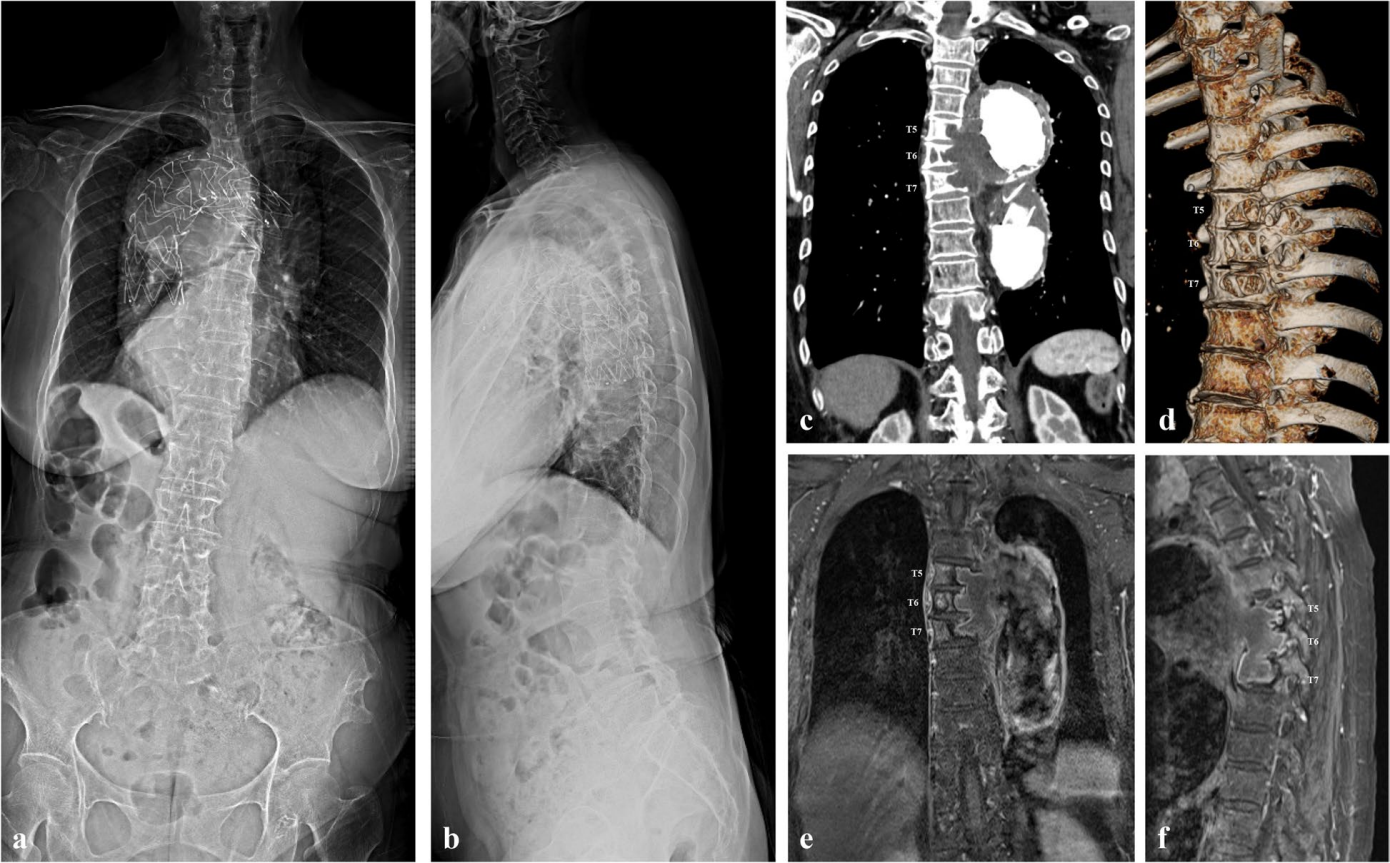
Initial presentation of this case. **a** and **b**, Plain radiographs revealing the implanted stent graft at the proximal descending thoracic aorta. **c** and **d**, Computed tomography of the aorta and thoracic spine show bony erosion into the T5–T7 vertebral bodies. **e** and **f**, Magnetic resonance imaging of the thoracic spine confirms a perianeurysmal outpouching lesion eroding into the T5–T7 vertebral bodies due to pulsating pressure

A 77-year-old female with a history of TEVAR (A 40 mm × 40 mm × 200 mm sized thoracic stent graft was deployed via right femoral artery access after anastomosis for left subclavican artery and left common carotid artery using direct suture without graft) due to descending thoracic aortic arch aneurysm 4 years ago was referred from the cardiovascular clinic to the spine center. She presented with persisting back pain, which began 3 years after TEVAR and progressively worsened. Physical examination was notable for tenderness in the upper thoracic region without any neurological deficits. Furthermore, no evidence of any suspected infection such as fever, elevated C-reactive protein, erythrocyte sedimentation rate, and procalcitonin was observed. Plain radiographs detected the previously implanted stent graft at the proximal descending thoracic aorta (Fig. 1a and b). Computed tomography (CT) of the aorta and thoracic spine showed bony erosion into the T5–T7 vertebral bodies (Fig. 1c and d). Magnetic resonance imaging (MRI) of the thoracic spine confirmed a perianeurysmal outpouching lesion eroding into the T5–T7 vertebral bodies due to pulsating pressure (Fig. 1e and f).

**Fig. 2:**
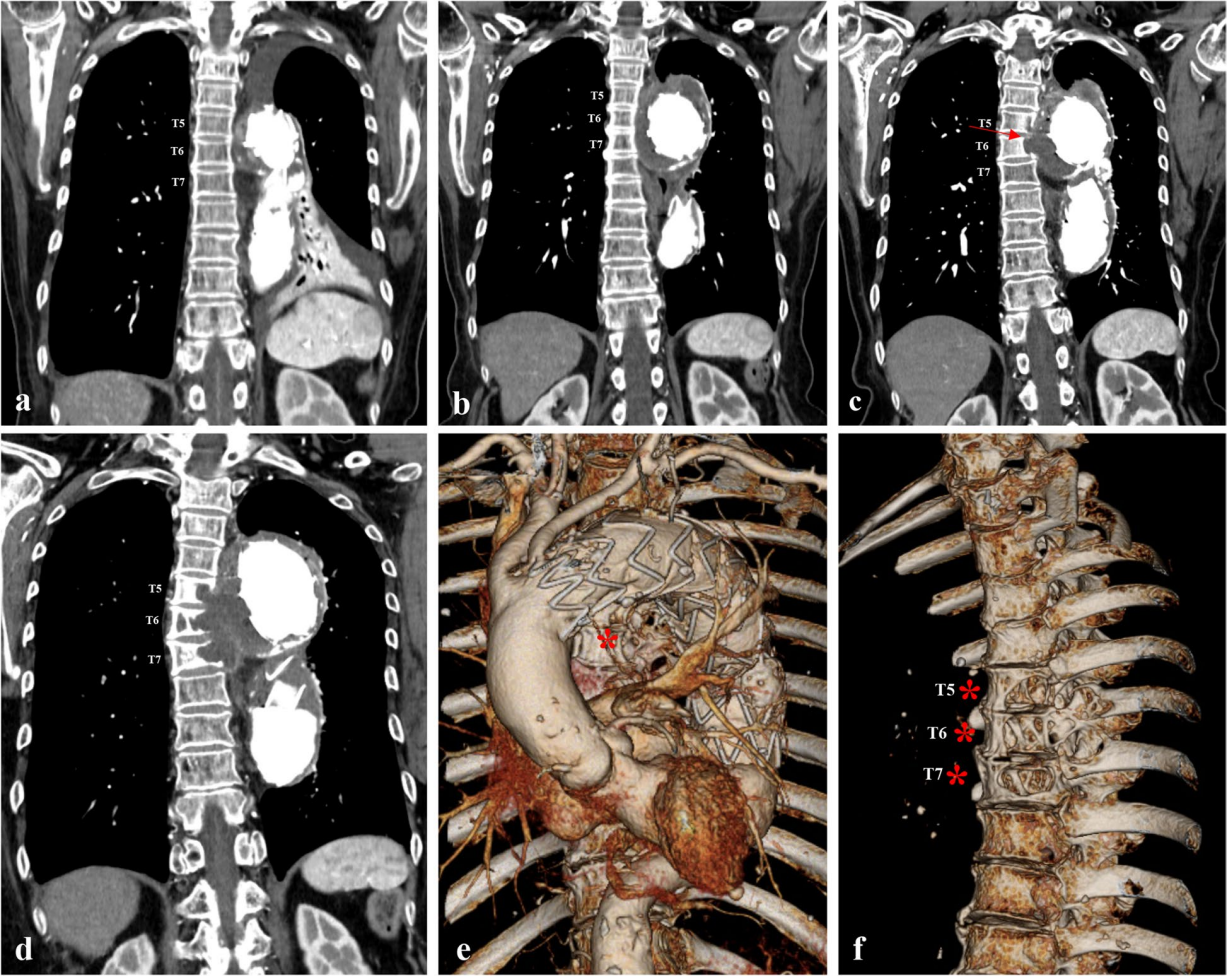
Progression of the thoracic vertebral body erosion in this case. **a**, Immediately after thoracic endovascular aortic repair (TEVAR), there was no contact between the thoracic aorta lesion and the thoracic vertebral body. **b**, At a 1.5-year follow-up visit after TEVAR, computed tomography (CT) revealed an increased size of the descending aorta, which was in contact with the thoracic vertebral body. **c,** At a 3-year follow-up visit after TEVAR, CT showed the perianeurysmal outpouching lesion involving T5 and T6. **d**–**f**, At a 4-year follow-up visit after TEVAR, CT revealed a more aggravated perianeurysmal outpouching lesion, and three-dimensional reconstruction of CT data revealed that an anterolateral portion of the thoracic vertebra body had eroded at T5–T7

The progression of the thoracic vertebral body erosion is presented in Fig. 2. After TEVAR procedure, there was no contact between the thoracic aorta lesion and the thoracic vertebral body (Fig. 2a). At a 1.5-year follow-up visit after TEVAR, CT revealed an increased size of the descending aorta, which was in contact with the thoracic vertebra body (Fig. 2b). At a 3-year follow-up visit after TEVAR, CT showed a perianeurysmal outpouching lesion involving T5 and T6 (Fig. 2c). At a 4-year follow-up visit after TEVAR, CT revealed a more aggravated perianeurysmal outpouching lesion (Fig. 2d), and three-dimensional CT reconstruction revealed that an anterolateral portion of the thoracic vertebra body was eroded at T5–T7 (Fig. 2e and f). Surgical treatment for a perianeurysmal outpouching lesion eroding the thoracic vertebra is required to prevent life-threatening conditions. However, considering the patient’s old age and high level of perioperative risk, our team decided to treat her conservatively for three months with monitoring of the perianeurysmal outpouching lesion.

**Fig. 3:**
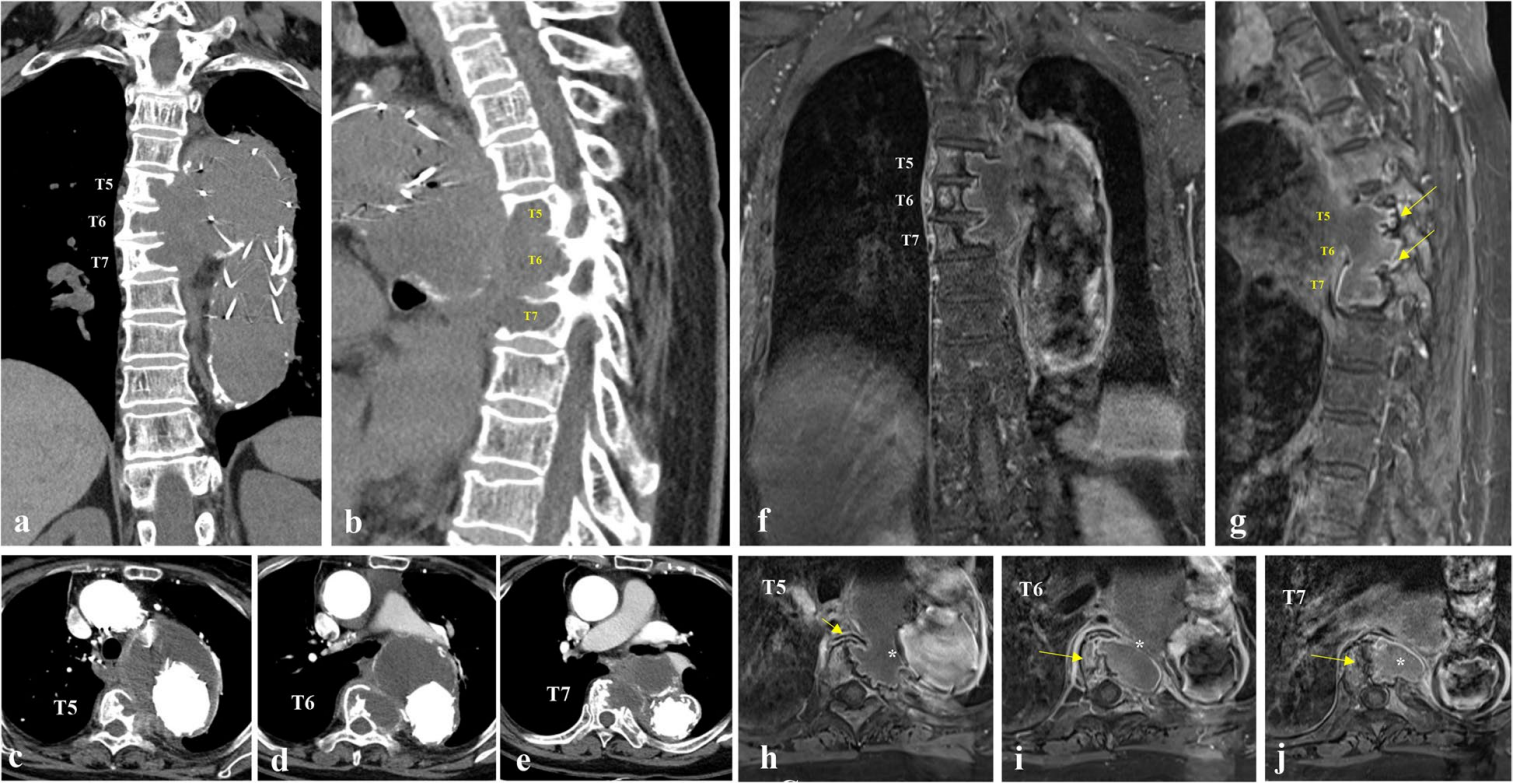
Aggravation of lesion at 3-month follow-up in this case. **a**–**e**, Three months later after surgery, follow-up computed tomography of the aorta showed an aggravated perianeurysmal outpouching lesion at the proximal descending thoracic aorta eroding into the T5–T7 vertebral bodies and adjacent left sixth and seventh ribs due to pulsating pressure from the complicated thoracic aortic aneurysm. **f**–**j**, Magnetic resonance imaging showed left neural foramen involvement at T5–T6 and T6–T7 and erosion of more than half of the vertebral body at T6 and T7

**Fig. 4:**
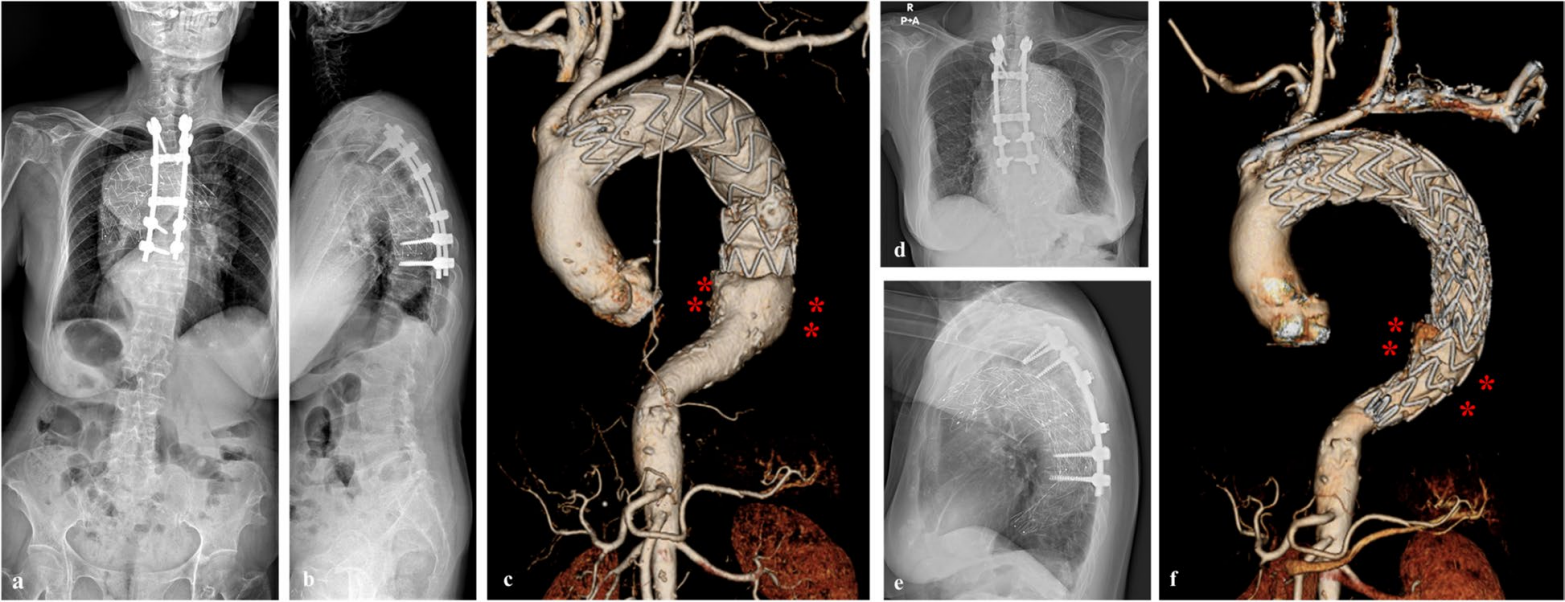
Surgical treatment in this case. **a** and **b**, Considering the rapid progression of this disease, posterior instrumentation and fusion from T3–T9 were performed to support the stability of the thoracic spine. **c**, Follow-up computed tomography of the aorta after posterior fusion showed remnants of the perianeurysmal outpouching lesion from endo-leakage after thoracic endovascular aortic repair (TEVAR). **d**–**f**, TEVAR was performed and significantly decreased the lesion remnants

Three months later, follow-up CT of the aorta showed an aggravated perianeurysmal outpouching lesion at the proximal descending thoracic aorta eroding into the T5–T7 vertebra bodies and adjacent to the left sixth and seventh ribs due to pulsating pressure from the complicated thoracic aortic aneurysm, presenting the type 1b of endoleak (Fig. 3a–e). MRI showed left neural foramen involvement at T5–T6 and T6–T7 (Fig. 3f and g) and erosion of more than half of the vertebral body at T6 and T7 (Fig. 3h–j). We initially planned for open surgery for the aneurysmal sac but the patient declined the extended and high-risk operation. Given the ongoing vertebral erosion, pedicle screw instrumentation and fusion (with resected autogenous bone and demineralized bone matrix) from T3–T9 by posterior approach were performed to support the stability of the thoracic spine, as half of the vertebral body was preserved (Fig. 4a and b). A follow-up CT scan of the aorta after posterior fusion revealed remnants of the perianeurysmal outpouching lesion. (Fig. 4c). The size of the lesion has not changed since posterior instrumented fusion. TEVAR was performed (Fig. 4d and e) and significantly decreased the lesion remnants (Fig. 4f). For the second TEVAR procedure via the right femoral artery, the bottom-up technique was applied; A 34 mm × 30 mm × 150 mm sized thoracic stent graft deployed distally first, and the larger endoprosthesis (40 mm × 40 mm × 200 mm sized thoracic stent graft inserted proximally into the smaller to facilitate good sealing. The patient’s symptoms and signs including tenderness were significantly improved at postoperative 3 months follow-up.

## Discussions

TEVAR has become the most common surgical procedure for pathology of the descending thoracic aorta [2]. For the treatment of thoracic aortic aneurysms, this minimally invasive technique has replaced traditional open surgical approaches due to its safety and efficacy benefits [2, 10]. TEVAR has an excellent success rate of up to 98% with low rates of perioperative mortality and morbidity [10, 11]. There are reports of post-implantation graft-related complications after TEVAR, but most are fairly uncommon; for example, incidence rates of 1% to 2.8% for device migration and 3.9% to 15% for endo-leakage have been noted [11–13]. Our rare case was caused by the endo-leakage as a post-implantation complication after TEVAR. By considering the no size change after re-TEVAR, we suspected that the endo-leakage was caused by disconnection of the stent graft, suggesting the type 1b. Furthermore, this case suggests that neglected endo-leakage after TEVAR can potentially result in large, complicated perianeurysmal outpouching lesions and subsequent vertebral erosion.

Vertebral erosion due to compression of the aortic lesion is very rare, with only cases reported [3–9]. Some of these cases involved vertebral body erosion caused by chronic contained descending thoracic aortic aneurysm ruptures involving thoracolumbar or lumbar lesions [3, 4, 6–8]. One case, reported by Luehr et al., involved thoracic vertebral body erosion at T7 with a complaint of back pain during a 10-year follow-up after TEVAR, which was treated by open thoracic surgery [5]. Furthermore, Wansink et al. reported a case of well-corticated vertebral body erosion at T10 and T11 involving about one-third of the vertebral body [9]. The patient was prone to vascular problems, with a history of hypertension, previous open repair of an aortic infrarenal aneurysm, and end-stage renal disease [9]. Contrary to these cases, our rare case was incidentally discovered due to the presence of asymptomatic and huge multi-level thoracic vertebral erosions, and successfully was treated with a re-trial of TEVAR and posterior instrumentation and fusion from T3–T9. Although there is a possibility of spinal ischemia in our case, the involvement of vertebral erosion didn’t infiltrate up to two-third of the vertebral body. We believe that the preservation of one-third of the vertebral body can reduce the risk of spinal ischemia, but long-term follow-up will be required. On the other hand, had more detailed images, such as intra-operative views in vascular approach, been presented and clinical outcomes measured and reported, the case study could have been strengthened. However, this represents a limitation of our report.

The most common site of vertebral body erosion is thoracolumbar and/or lumbar lesions caused by infra-renal aortic aneurysms [4–6, 8, 9]. However, in the case of TEVAR endo-leakage, the thoracic level is most susceptible to vertebral erosions [5, 9]. Compared to thoracolumbar and lumbar lesions, the thoracic vertebrae have less mobility and are contained by the ribs, increasing the potential for asymptomatic progression. Considering our rare case, thoracic vertebral erosion can potentially occur without symptoms after TEVAR surgery, so routine and serial CT follow-up is essential to prevent catastrophic outcomes.

Thoracic vertebral erosion is a rare and fatal complication after TEVAR [5, 9]. Considering the rapid progression of vertebral erosion, it is essential to include infection (infected aneurysm or infected TEVAR) in the differential diagnosis. However, as previously mentioned, no evidence of any suspected infection was observed. In this case, the erosion originated from the expanding aneurysm and not from the TEVAR device itself. The suggested mechanism of vertebral erosion is initially mediated by the formation of a perianeurysmal outpouching lesion from endo-leakage [2]. The size of this lesion gradually increases and eventually contacts the adjacent vertebral body [5, 9]. The repetitive mechanical pressure from large-sized pulsatile perianeurysmal outpouching lesions causes ischemia on the vertebra body, which subsequently leads to lysis and bony destruction [9]. Therefore, delayed diagnosis of vertebral body erosion, like in our case, results in a high level of perioperative risk and morbidity, and initial vascular treatment for endo-leakage is important through serial follow-up.

There are few reports of surgical treatment of spinal complications related to aortic aneurysms [4, 8]. In particular, a case with management of both vertebral erosion and graft failure has not been reported. Posterior instrumented fusion is used to support the stability of the spine for vertebral erosion [4, 14]. Meanwhile, open surgical repair with graft removal is the gold standard after mechanical failure of TEVAR [5]. Although open surgical repair is the first choice in the case of mechanical failure of TEVAR, the high risk of mortality for open surgical repair made this decision difficult. In this case, we chose a two-stage operation plan: first, posterior instrumentation and fusion were performed to support the stability of the thoracic vertebra; then, TEVAR for a perianeurysmal outpouching lesion was successfully completed [15, 16]. In our case, we initially planned an open surgical procedure for the aneurysmal sac, but the patient declined due to the extended and high-risk nature of the operation. Given the ongoing vertebral erosion, we prioritized restoring spinal stability through posterior instrumented fusion. Although there is no established surgical indication, addressing the vascular issue prior to spinal instrumentation would be the most ideal approach. However, in our case, the patient initially declined to perform vascular procedures due to high risk involved. Despite many potential risks and persisting status of the endo-leakage, we were able to restore spinal stability through posterior instrumented fusion, as half of the vertebral body was preserved. Furthermore, to reduce catastrophic bleeding during pedicle screw instrumentation, we used short-size pedicle screws or skipped in areas with significant vertebral erosion. Therefore, an interdisciplinary treatment arrangement involving the spine surgeon and vascular team is important in cases with a high index of suspicion from closed observation. Furthermore, our case provides that a minimally invasive vascular approach is one of the useful treatments of choice for cases with a high risk of mortality after mechanical complications of TEVAR.

In conclusion, our case presented thoracic vertebral body erosion due to a perianeurysmal outpouching lesion after TEVAR. Since progression of such an event can lead to a catastrophic outcome, and because treatment options vary, serial follow-up through an interdisciplinary approach is important in cases with a high index of suspicion of such a lesion.

## Data Availability

The data that support the findings of this study are not openly available due to reasons of sensitivity and are available from the corresponding author upon reasonable request.

## Abbreviations

TEVAR: Thoracic endovascular aortic repair
CT: Computed tomography
MRI: Magnetic resonance imaging
